# Self-reported cycling behavior and previous history of traffic accidents of cyclists

**DOI:** 10.1186/s12889-024-18282-7

**Published:** 2024-03-13

**Authors:** Enayatollah Homaie Rad, Fatemeh Kavandi, Leila Kouchakinejad-Eramsadati, Kamran Asadi, Naema Khodadadi-Hassankiadeh

**Affiliations:** 1 Social Determinants of Health Research Center, Trauma Institute, Guilan University of Medical Sciences, Rasht, Iran; 2 School of Medicine, Guilan University of Medical Sciences, Rasht, Iran; 3 Guilan Road Trauma Research Center, Trauma Institute, Guilan University of Medical Sciences, Rasht, Iran; 4 Orthopaedic Research Center, Department of Orthopaedic Surgery, School of Medicine, Poursina Hospital, Guilan University of Medical Sciences, Rasht, Iran; 5 Guilan Road Trauma Research Center, Trauma Institute, Poursina Hospital, Namjoo St, 4193713194 Rasht, Guilan Iran

**Keywords:** Cycling, Safety, Traffic accident, Injury, Trauma, Behavior

## Abstract

**Background:**

Cyclists are vulnerable traffic users and studying the cycling behavior of professional and elite cyclists, their previous history of traffic accidents combined with the current knowledge on high-risk behaviors of this group can be a useful basis for further studies on ordinary cyclists. This study aimed to determine the relationship between cycling behavior and the previous history of traffic accidents among members of the Cycling Federation of Guilan province in 2022.

**Methods:**

A descriptive-analytical study was performed in which the Bicycle Rider Behavior Questionnaire (BRBQ) constructed in the *Porsline* platform was distributed using the WhatsApp social network. All participants were asked to self-report their cycling behavior. The final analysis was performed by using STATA software (version 14).

**Results:**

The study subjects included a total of 109 cyclists with a mean age of 38.62 ± 10.94 years and a mean cycling experience of 13.75 ± 11.08 years. Using the logistic regression model, the relationship between gender (*P* = 0.039), years of cycling experience (*P* = 0.000), and education level (*P* ≤ 0.00), with previous traffic accidents, was found significant. There was also a significant relationship between stunts and distractions (*P* = 0.005), signaling violation (*P* = 0.000), and control error (*P* = 0.011) with previous traffic accidents. A significant association existed between stunts and distractions (*P* = 0.001) and signaling violation (*P* = 0.001) with a previous history of traffic injury within the last 3 years.

**Conclusions:**

The findings of this study can be used to establish cyclist safety and preventative planning in society. In behavior change intervention programs, it is best to target male cyclists with higher-level education. In addition, the behavior of the cyclists whose predominant term of signaling violations must be corrected should be targeted. It is necessary to shape information campaigns and educational programs aimed for cyclists with common high-risk behaviors, especially signaling violations.

**Supplementary Information:**

The online version contains supplementary material available at 10.1186/s12889-024-18282-7.

## Introduction

The ever-increasing development of communities has led to a greater density of motor vehicles, traffic accidents and eventually increasing air pollution [1–3]. A proposed solution to address the shortcomings of the current transportation system is to increase the number of bicycle trips [2, 4, 5].

Cycling as an active mode of transportation has numerous health benefits and can help reduce the consequences of a sedentary lifestyle for the body such as cardiovascular diseases [6, 7], type 2 diabetes and obesity [8, 9], the risk of colon [10, 11] and breast cancer [12, 13], hypertension, hyperlipidemia [14], osteoporosis [10, 15], depression and anxiety [5, 16].

Although cycling is increasingly encouraged across society, concerns about cyclist’s safety are one of the main barriers to its adoption as a means of transportation [17–19]. Compared with car drivers, cyclists are much more likely to be seriously injured in car crashes [20, 21]. Between 2007 and 2016, the death rate of cyclists in traffic accidents increased by almost 20% [22].

Several factors influence the cyclist’s safety including environment and infrastructure [23, 24], exposure to unsafe cyclists [10, 25, 26], demographic characteristics [10, 17], socioeconomic status [27, 28], behavioral patterns [29, 30], and temporal factors [24, 31]. Each category contains several factors that can affect the number and severity of bicyclist’ accidents.

The efforts made for the prevention of traffic accidents among cyclists are based on the findings obtained from behavioral studies on these road users. The investigations suggest that cyclists have certain, different behaviors. They choose their cycling paths based on a number of criteria such as the length of the trip, the number of turns, the traffic volume, and slopes. If there are exclusive paths, cyclists prefer to use them on high-risk routes [32, 33]. However, some cyclists prefer commuting on street lanes to cycle-only paths because these lanes follow the main road lines and are more straight [34]. In a behavioral study, more than 90% of Chinese cyclists reported involvement in at least one crash (regardless of severity) with other road users, and 88.85% of the subjects had a previous history of an accident with pedestrians [35].

Individual, social, cultural, and environmental factors affect the behavior of cyclists. Individual (age, gender, race, income), psychological (pleasure, motivation, self-confidence), behavioral (cycling skill or experience), and environmental (institutional and school supports) [36].

Investigating the association between demographic factors and behaviors demonstrated that compared with female ones, male cyclists are more prone to traffic accidents regardless of age. In addition, road type, vehicle type, the maneuver of involved vehicles and cyclists, collision type, accident time, accident day, and accident season have proven to be different in men and women [37]. Examining the social factors and behaviors of cyclists revealed that when their perceived skill was low, they had more risky behaviors [38].

### Background

The analysis of statistics in the Iranian Legal Medicine Organization of Iran reported 526 deaths of cyclists between April 2019 and March 2011. On average, 188 people are killed each year in cycling accidents. Moreover, 93.5% of mortalities are caused by the collision between bicycles and motor vehicles [39].

Guilan province, located on the edge of the Caspian Sea with a population of about 2.4 million people and an area of 14,700 square kilometers, is among the top three provinces of Iran in terms of traffic accident fatalities [40].

It is one of the tourist attractions located in the north of Iran. Bicycle accidents has been increasing in Guilan due to the increase in travel from other provinces to this area and the high bicycle traffic volume [41].

Cycling Federation of Guilan has 22 professional members and an international track for cycling plus other suitable facilities for holding cycling competitions and camps.

Few studies have been conducted to investigate the behavior of cyclists who were members of the cycling federation. The behavior of these cyclists and their history of traffic accidents should be examined closely, considering that previous studies have had contradictory findings in this regard. The present scrutiny can fill the gap that exists in the knowledge of traffic safety among road users with risky behaviors, especially in local settings.

### BRBQ

The BRBQ, which is used to evaluate the behavior of cyclists is a little different in terms of the number of questions and dimensions in various countries. For instance, the Chinese version of the BRBQ has four subscales and 35 questions. The first subscale, which is “rule and aggressive violations”, has 9 questions (riding a bicycle while drunk, changing lanes without observing the road conditions, making a sudden turn) and aggressive violations (making rude gestures if angry with the behavior of other road users). The second subscale is “Ordinary violations”: and includes 6 questions that are common among Chinese cyclists. The third subscale is “personal control errors” and includes 5 practical errors, and the fourth subscale includes 5 questions related to distracting behaviors [35]. A simpler version of the questionnaire with 18 questions has also been validated in China, including 6 violations, 6 errors, and 6 positive behaviors, based on a 5-point Likert response (0 = never, 1 = rarely, 2 = sometimes 3 = often, 4 = always) [42].

Recently, the Iranian version of this questionnaire has been validated. It was found that the Iranian version of BRBQ has practical psychometric properties and has good validity and reliability [43]. Considering the characteristics of the instrument used it should be noted that it was a 34-item questionnaire (BRBQ) valided by 306 Iranian cyclists. This questionnaire has five subscales, which are “Stunts and Distractions”, “Traffic Violations”, “Notice Failures”, “Control Errors”, and “Signaling Violations” [43]. The Iranian version of BRBQ was prepared in three stages. The first step of its preparation was based on the MRBQ questionnaire Elliott, Baughan et al. (2007) from which similar misbehaviors among bicyclists and motorcyclists were extracted. It is a five-factor questionnaire with 43 items and a five-point Likert scale (1: never, 5: almost always). Its reliability coefficient was 0.84 in the traffic errors factor with Cronbach’s alpha, 0.87 in the speed violation factor, 0.81 in the risk-taking behavior factor, 0.70 in the safety equipment factor, and 0.73 in the control errors factor [44]. In the second step, the original version of the Driving Style Recognition Based on Driver Behavior Questionnaire (1990) was used on perception errors and violations (e.g. crossing a red light) [45]. Next, the available texts on safety were used to identify the inappropriate behavior of cyclists and develop a behavioral questionnaire with 39 questions on a five-point Likert scale (1: never, 5: almost always). Its sub-scales included a-driving violations with a 0.84 reliability rate b- stunts and distractions with a reliability rate of 0.79 c- control errors with a reliability rate of 0.77 d- notice failure with a reliability rate of 0.78 and e- signaling violations with a reliability rate of 0.76. The reliability of this questionnaire was in the range of 0.70 to 0.84 [43]. It has been reported that the average time for completing this questionnaire is approximately 6 min [46].

### Objectives

The objective of this study is to improve our knowledge of the demographic characteristics of cyclists, 5 subtypes of cyclists’ behaviors, their safety equipment, and the history of traffic accidents and injuries in the past three years and earlier, and their relation to the behaviors of cyclist.

## Methodology

### Study protocol

An online survey was done as a convenience. This study method was used in the past for BRBQ in electronic convenience form [30, 47]. The study protocol was reviewed by the Medical Ethics Committee of Guilan University of Medical Sciences and its compliance with the Declaration of Helsinki was confirmed (Code: IR.GUMS.REC.1399.536). However using *Porsline*, an online survey platform in Iran (https://survey.porsline.ir), a questionnaire was constructed exactly based on the BRBQ. The link to participate in the research was provided to the head of the Guilan Cycling Committee to be distributed among all professional and semi-professional members. The online survey link was active in the first six months of 2022 in Persian language. After that, the link was automatically disabled. After entering the link, the participants first read the objectives of the research and if they agreed to participate in the study, they answered the questions that met illegibility (being native and having at least one year of membership experience). The BRBQ was activated only for the participants who had inclusion criteria to enter the study. These strategies helped to maintain the voluntariness and confidentiality of the participants’ responses [47, 48]. Completing the questionnaire took about five minutes.

### Participants

The total population sampling used in this study included all 149 members of the Cycling Federation of Guilan province in the north of Iran who met the inclusion criteria. Consent to participate in the research was obtained before filling out the questionnaire. The participants were free to read all of the questions and write the requested information or if they were unwilling to do so, or changed their minds, could leave the questionnaire incomplete.

#### Inclusion criteria

The inclusion criteria were being a native and resident of Guilan, and having at least one year of professional and semi-professional membership in the Cycling Federation of Guilan. Cyclists who did not submit a completed questionnaire for any reason (*n* = 36) were excluded from the study.

### Sample

Of a total of 145 professional cyclists, 109 members of The Cycling Federation of Guilan participated in this study (75.1% response rate). In their self-reports, the mean age was 10,943 ± 38.62 and the mean cycling experience was 11,084 ± 13.75 years. The majority of the subjects were men (*n* = 68, 62.39%). Fifty-seven of the participants were married (52.78%), 66 had a job (60.55%) (Full-time and part-time jobs) and 59 (55.14%) had associate/bachelor degrees. Ninety-nine participants (90.83%) had a car/motorcycle driving license. Most of them (*n* = 67, 61.47%) stated sport and recreational purposes for cycling (Table 1).

**Table 1:** Socio-demographic variables of bicyclists (*n* = 109)

Variable	Categories	N (%)
Gender	Male	68 (62.39)
	Female	41 (37.61)
Marital Status	Single	51 (46.79)
	Married	57 (52.29)
	Divorced	1 (0.92)
Employment	Employed	66 (60.55)
	Others	43 (39.45)
Education	High school graduate/High school	21 (19.63)
	Associate/Bachelor	59 (55.14)
	Master	22 (20.56)
	Doctorate	5 (4.67)
Driving license	Yes	99 (90.83)
	No	10 (9.17)
Cycling purpose	Exercise/Recreation	67 (61.47)
	Professional	27 (24.77)
	Work/shopping/others	15 (13.67)

### Questionnaires

**A-** The demographic section includes questions about age, gender, marital status, employment, and education. Employment was divided into two types of having a job (governmental or non-governmental) and not having a job (others) such as being a housewife, unemployed, student, and soldiers passing their military service.

**B-** The bicycle use section contained questions about having a driving license (car or motorcycle), years of cycling experience, the main purpose of cycling (exercise/recreation, professional/racing, going to work/shopping, and household chores/others), wearing a helmet while cycling, wearing a helmet in the last cycling experience, having a bicycle equipped with night light reflectors, previous history of accident/injury in the last three years, previous history of bicycle accident/injuries more than three years ago, previous accident/injury as a driver, passenger, or pedestrian.

### Outcome measure

#### Travel behavior

We also obtained travel behavior by BRBQ. The validity and reliability of this questionnaire were confirmed by Hezaveh et al. (2018) in a study on novice, semi-professional and professional members of the Cycling Federation of Iran. The participants were asked about the average amount of involvement in each of the following behaviors while riding a bicycle in the last month. Five behavioral dimensions were examined. The first dimension included 14 violations, called “traffic violations” such as riding two-way cycle tracks or riding a bicycle outside the designated bicycle lanes, etc. The second dimension had 9 items related to carelessness such as listening to music or riding a bicycle under the influence of alcohol and drugs, etc., which was specified as “stunts and distractions”. The third dimension included 8 items related to bicycle control such as difficulty in controlling the bicycle at the lower end, which was called “control errors”. The fourth dimension was related to cyclists’ lack of attention to other road users, which was called “notice failures”. In the fifth dimension, there were 3 violations related to communication with other road users, which was called “signaling violation” (Appendix 1).

### Statistical analyses

Frequency and percentage were used to report the demographic variables and the way the bicycle was used. Mean and standard deviation were also used for the variables of age, years of cycling experience, and dimensions of cycling behavior. We used 12 covariables in the study. For the dependent variable (cycling behavior), the logistic regression model was used. All of the analyses were performed with Excel and STATA software (version 14).

## Results

The majority of the participants (*n* = 85, 77.98%) wore a helmet, 77 (71.64%) had used a helmet in their last ride, and 84 (77.06%) had reflectors on their bikes (Table 2).

**Table 2:** Safety equipment used by the cyclists (*n* = 109)

Variable	Categories	N (%)
Helmet	Yes	85 (77.98)
	No	24 (22.02)
Helmet use in the last ride	Yes	77 (71.64)
	No	32 (29.36)
Reflectors	Yes	84 (77.06)
	No	25 (22.94)

Most of 57 cyclists (52.29%) had no history of traffic accidents during the last 3 years and the rest had 1.512 ± 1.091 times crashes during the last three years. Fifty-six people (51.38% within the last 3 years) did not have any injuries and the rest were injured 1,461 ± 1,045 times on average during the last 3 years (Table 3).

**Table 3:** Characteristics of traffic accidents in the cyclists (*n* = 109)

Variable	Category	N (%)
Previous accidents in the past three years	None	57 (52.29)
	One	22 (20.18)
	Two	11 (10.09)
	Three	8 (7.34)
	Four	4 (3.67)
	Five and more	7 (6.42)
Previous injuries in the past three years	None	56 (51.38)
	One	25 (22.94)
	Two	12 (11.01)
	Three	6 (5.50)
	Four	3 (2.75)
	Five and more	6 (6.42)
History of accidents more than three years ago	Yes	57 (52.29)
	No	52 (47.71)
History of injuries more than three years ago	Yes	52 (47.71)
	No	57 (52.29)
Experience of accidents as pedestrian/passenger/driver	Yes	45 (41.28)
	No	64 (58.72)
Experience of injuries as a pedestrian/passenger/driver	Yes	38 (34.86)
	No	71 (65.14)

The mean of BRBQ dimension included signaling violation 56.80 ± 27.57, notice failure 25.96 ± 13.80, control errors 19.55 ± 11.31, stunts and distractions 18.02 ± 12.40, and traffic violations 15.28 ± 11.72.

### A- The relationship between demographic characteristics and BRBQ dimensions with history of traffic accidents

According to the logistic regression model, the relationship between gender and the previous history of traffic accidents was significant (*P* = 0.039). It can be said that women had fewer crashes during the last 3 years. There was also a significant association between the years of cycling experience and the previous history of traffic accidents (*P* = 0.000), that is, the likelihood of traffic accidents increased with more years of cycling experience. Education was significantly associated with previous history of traffic accidents (*P* ≤ 0.00) and the higher the education was, the higher the number of crashes were. No significant relationship was observed between the purpose of cycling, marital status, and employment with previous history of traffic accidents.

Regarding the dimensions of BRBQ, a significant relationship was observed between stunts and distraction (*P* = 0.005), signaling violation (*P* = 0.000), control errors (*P* = 0.011), and previous history of traffic accidents. Therefore, as the number of stunts and distractions increases, there will be more traffic accidents. Besides, when bicycles are more equipped with reflectors, the probability of crashes during cycling decreases, and with more control errors, more crashes occur (Table 4).

**Table 4:** Logistic regression of the relationship between BRBQ and history of traffic accidents

Traffic accidents in the past three years	Coef	Std. Err.	z	P>|z|	[95% Conf. Interval	
					Low	Up
Age	0.003	0.016	0.19	0.848	− 0.035	0.028
2. Gender	− 0.590	0.286	-2.06	0.039	-1.152	− 0.029
Cycling duration	0.007	0.001	4.07	0.000.	0.003	0.010
Cycling purpose						
Exercise/Recreation	− 0.349	0.376	-0.93	0.353	-1.087	0.387
Professional	− 0.065	0.381	-0.17	0.863	− 0.813	0.681
Education						
Associate/license	0.843	0.401	2.10	0.036	0.056	0.056
Master Degree	1.246	0.412	3.02	0.002	0.438	2.055
Doctorate Degree	1.006	0.681	1.48	0.140	− 0.328	2.342
1.Marraid	0.370	0.246	-1.50	0.133	− 0.854	0.113
1.Employed	− 0.251	0.244	-1.03	0.303	− 0.729	0.227
Stunts and distractions	0.031	0.010	2.83	0.005	0.009	0.052
Signaling violation	− 0.016	0.004	-3.52	0.000	− 0.025	− 0.007
Attention failures	− 0.010	0.010	-1.05	0.293	− 0.030	0.009
Traffic violations	− 0.0159	− 0.011	1.34	0.179	− 0.0392	− 0.007
Control error	0.033	0.012	2.55	0.011	0.007	0.058

### B- Examining the relationship between BRBQ dimension and history of traffic injury

According to the logistic regression model, there was a significant relationship between stunts and distractions (*P* = 0.001) and signaling violation (*P* = 0.001) and history of traffic injury in the last 3 years, so that the higher the number of stunts and distractions, the more likely the injuries were to occur. The more signals and signs cyclists used, the lower the amount of injuries were (Table 5).

**Table 5:** Logistic regression of the relationship between BRBQ and history of traffic injury

Injury in the past three years	Coef	Std. Err.	z	P>|z|	[95% Conf. Interval	
					Low	Up
Age	− 0.005	0.014	-0.39	0.698	− 0.034	0.023
2. Gender	0.281	0.258	1.09	0.276	− 0.224	0.787
Cycling duration	0.000	0.002	0.28	0.783	− 0.004	0.006
Cycling history						
Exercise/Recreation	− 0.189	0.362	-0.52	0.601	− 0.901	0.521
Professional	0.249	0.383	0.65	0.516	− 0.502	1.001
Education						
Associate/Bachelor	0.100	0.349	0.29	0.773	− 0.584	0.785
Master	0.571	0.349	1.63	0.103	− 0.114	1.256
Doctorate	− 0.508	0.810	-0.63	0.530	-2.096	1.079
1. Married	0.249	0.240	1.04	0.298	− 0.221	0.720
1.Empolyed	− 0.199	0.238	-0.84	0.401	− 0.666	0.266
Stunts and Distractions	0.037	0.010	3.41	0.001	0.015	0.058
Signaling violation	− 0.014	0.004	-3.22	0.001	− 0.023	− 0.005
Notice Failures	− 0.015	0.010	-1.48	0.139	− 0.036	0.005
Traffic Violations	− 0.007	0.011	-0.60	0.548	− 0.030	0.016
Control Error	0.020	0.012	1.56	0.119	− 0.005	0.045

## Discussion

In the present study, almost a quarter of cyclists reported previous experience of one accident and one injury in the past three years. This was less than half of the reports of other similar studies conducted on ordinary cyclists. In a study conducted in Iran, more than half of the respondents reported at least one bicycle crash [43]. In O’hern’s study, almost half of the participants reported previous accidents including minor crashes, while riding a bicycle in the last two years [49]. In Wang-zhang’s study, 88.85% of the subjects stated that they had been involved in at least one pedestrian crash. Moreover, 16.09% were involved in at least one crash with a motorized vehicle, and 67.46% were involved in at least one crash with another non-motorized vehicle [35].

### The effect of gender on traffic accident history

As the results of the present study showed the relationship between gender and history of traffic accidents was such that women had significantly fewer crashes during the past three years. In a study in northern Iran, the behavior of pedestrians was related to gender, and women had less risky behavior [50]. In a study by Xiaomeng Li (2022) comparing three countries, male cyclists were more risk-averse than female cyclists and reported more crashes (19). In Hezaveh’s (2018) study, stunts and distractions were more in male than female cyclists. Conversely, female cyclists reported more control errors than male cyclists [43]. In Useche’s (2018) study, the rate of violations committed by men was significantly higher and they scored lower in positive behaviors than female cyclists while the scores in cycling violations were not significantly affected by the gender of the participants [51]. The results manifested that the female members of the federation were more cautious than men and had fewer crashes.

### The effect of cycling years on the history of traffic accidents

In the present study, the number of traffic accidents increased with mean cycling history. However, in another study, the bivariate correlation between dimensions and cycling experience indicated that with increasing years of experience, the abnormal behaviors of cyclists decreased [43]. In another study, cyclists who cycled five hours or more per week reported significantly lower scores for violations, while scores for errors and positive behaviors were not affected by the years of cycling. Experience not only makes people perceive less risk but also acts as positive reinforcement [42]. It is difficult to determine whether experience leads to a perception of risk closer or farther from the actual risk [52].

### The relationship between cyclist education and traffic accident history

In the present study, the history of traffic accidents was significantly more among cyclists with higher levels of education. than in those under this level. It has been reported that cyclists with a university education and more knowledge of traffic issues had more risky behavior [53]. Some researchers also stated that the higher the level of education of cyclists, the more they tend to violate traffic laws [54]. Though, when the control of risky behavior was not related to higher levels of education, the quality of traffic education during training is low. These findings are incompatible with the result of an Iranian study. First, traffic and driving training is barely taught at schools. Second, the trained materials are not logically coherent. They are included in textbooks in a haphazard fashion without any logical purpose [55]. Needless to say, familiarity with traffic regulations is very important [56]. It was previously reported that requiring e-bike users to participate in a training program and pass certain tests can be an effective way to reduce their tendency to skip red lights [57]. Therefore, when developing educational or certification programs, more attention should be paid to certain groups and their safety knowledge [53]. Educational and behavioral interventions should be strengthened to reduce the dangerous behaviors of cyclists [58]. Safety training for users should be developed in addition to designing novel safety facilities for cyclists [59]. In big cities, there is a need to knowledge and improve the safety of cyclists because they commit more risky behaviors [60].

### The most common behaviors of cyclists

In the present study, the most common behavior among the five investigated dimensions is signaling violations, followed by attention failure, control errors, stunts and distractions, and traffic violations. They ranked second to fifth in terms of frequency respectively. According to the literature, risky/positive behaviors and personality traits play a role as key factors in making cyclists safer [61, 62]. In a study, the total positive behaviors of cyclists were more than risky behaviors [47].However behavioral patterns are largely dependent on demographic variables and indicators of each country. In one study, the most common responses were “forgotten turn signal” and “turning onto the sidewalk.” Other violations commonly included “cycling after drinking,” “using a cell phone while cycling,” “skipping red light,” and “cycling at night without front or rear lights” [49]. In an Iranian study, not using hand signals to communicate with other road users was reported as one of the common behaviors. Distraction by music and mobile phones, as well as aggressive violations towards other road users, were among the most common behaviors. Driving under the influence of alcohol and drugs was one of the least reported behaviors [43]. Therefore, the most frequent behaviors (wrong signaling) in our study were similar to other studies. This means that the most basic training, which is hand signal training, is not given enough training to these members, or their compliance and implementation are not monitored enough.

### Relationship between the different dimensions of BRBQ and previous experience of traffic accidents and injury

Considering the different dimensions of BRBQ, there was a significant relationship between stunts and distractions, signaling violations, and controlling errors with previous traffic accidents in the past three years. According to the present study, a significant relationship existed between stunts and distractions and signaling violations, with previous traffic injuries in the past three years. In Hezaveh’s study, stunts and distraction, and traffic violations had a positive and significant correlation with reported multiple-vehicle accidents [43]. In Wang-zhang’s study, aberrant behaviors and aggressive offenses, habitual offenses, and distraction were significantly associated with all crashes reported in the past three years, and among all aberrant behaviors, habitual offenses were the strongest factor in all traffic accidents [35].

### Limitation

One of the limitations of this study was the few number of studies conducted in the world on this issue with most of them investigating the behavior of ordinary cyclists. The study on the behavior of professional cyclists makes it difficult to generalize the results to other ordinary cyclists in Guilan province, but the results obtained were contrary to the expectations of the results of studies in other countries. This means that the behavior of cyclists who are members of the federation can be similar to ordinary cyclists in the world. In addition, the results of this study revealed that BRBQ is a useful instrument for specifying the types of high-risk behaviors of cyclists. Another limitation was the small sample size, however, the entire population of the federation members entered the sampling process, and a three-fourths participation rate is considered a favorable response in questionnaire studies. Likely, some cyclists could not remember the accurate details of their previous accidents, especially minor crashes or the ones that occurred a long time ago (historical bias). Some cyclists might have reported only serious crashes and injuries, not any type of traffic accident (with any degree or severity). Using a self-report questionnaire can also be considered a limitation since cyclists’ reports of their behavior can be different from their actual behavior observed while cycling.

## Conclusion

The results of the present study can create a new understanding of different dimensions of the cycling behavior of cyclists who are members of the cycling federation in Guilan province, Iran. The study can provide insights on which behavioral patterns of cyclists might affect the outcome of cycling. Significant and unexpected levels of risky behavior were observed among cyclists in this study, which were significantly connected with their history of previous traffic accidents. Therefore, it is necessary to emphasizeprofessional cycling behavior and adherence to cycling rules and regulations in this group of road users. The findings of this study can be used to promote cyclists’ safety and preventative planning. In behavior change intervention programs, it is best to target male cyclists with higher levels of education. In addition, the behavior of cyclists whose predominant term of signaling violations must be corrected can be targeted. It is necessary to conduct information campaigns and educational programs for cyclists with common high-risk behaviors, especially signaling violations.

### Electronic supplementary material

Below is the link to the electronic supplementary material.

Supplementary Material 1

## Data Availability

All data generated or analyzed during this study are included in the submission.
