# Supplementary material for: Self-reported cycling behavior and previous history of traffic accidents of cyclists
Source: BMC Public Health. 2024 Mar 13;24:780. doi: 10.1186/s12889-024-18282-7 (PMC10936005; doi:10.1186/s12889-024-18282-7)
Supplement: Supplementary file 1 — Supplementary Material 1 [file 12889_2024_18282_MOESM1_ESM.pdf]

Appendix-1

Cycling behavior

On average, in the past month, how often have you performed each of the following behaviors while riding a bicycle?

(Never, Rarely, Sometimes, Often, Almost, Always)

**Behavior**	**Subscale**
1. Not noticing pedestrians when turning.	Notice Failures
2. Not noticing a pedestrian coming out from behind a parked car.	Notice Failures
3. Not noticing the pedestrian who is waiting to cross the street on the crosswalk.	Notice Failures
4. Ignoring the right of way sign and the possibility of crashes with a car that has the right of way.	Traffic Violations
5. Being distracted or busy with something in such a way that you have to brake hard to avoid crashing into the car in front that has slowed down.	Notice Failures
6. Riding a bicycle at the speed of traffic, in such a way that it is difficult for you to stop behind a traffic light.	Control Error
7- Increasing the speed to pass the traffic light that is about to turn red.	Traffic Violations
8- Crossing the red light.	Traffic Violations
9. Riding a bike at high speed in a corner, in such a way that you feel like you are losing control.	Control Error
10- Racing with other cyclists, motorcyclists, or drivers.	Stunts/Distractions
11- Cycling in the fast lane of the street.	Traffic Violations
12- Riding a bicycle at a close distance to the car in front so that it is difficult to stop in an emergency.	Control Error
13- Getting tired of other drivers, motorcyclists, pedestrians, and cyclists.	Notice Failures
14- Get angry with other road users (cyclists, pedestrians, and drivers) and become aggressive towards them.	Traffic Violations
15- Showing your anger towards others by any means possible.	Traffic Violations
16- Talking on the phone while cycling, even using Bluetooth.	Stunts/Distractions
17- Texting while cycling.	Stunts/Distractions

18- Cycling under the influence of alcohol or drugs.	Stunts/Distractions
19- Listening to music while cycling.	Stunts/Distractions
20- You don't know what gear to use while riding a bicycle.	Control Error
21- Sliding with a bicycle when the street surface is slippery.	Control Error
22. Get angry with other road users (cyclists, pedestrians, drivers) and become aggressive towards them.	Control Error
23- Unicycle riding.	Stunts/Distractions
24- Maneuver between cars.	Stunts/Distractions
25- Losing control of the bicycle due to an obstacle in the street (such as a sewer valve) or road pavement failure.	Control Error
26- Difficulty controlling the bike downhill.	Control Error
27- Cycling against the direction of traffic flow.	Traffic Violations
28- Not giving the right of way to pedestrians you notice.	Traffic Violations
29- Bicycling outside the bicycle lanes (when these lanes are available on the street).	Traffic Violations
30- Cycling in areas where the passage of bicycles is prohibited (such as highways, sidewalks, and special lanes for express buses...).	Traffic Violations
31- Bicycling in two-track mode.	Traffic Violations
32- Signaling with the hand when changing direction (turning, overtaking, etc.)	Signaling violation
33- Signaling with hand while braking.	Signaling violation
34- Ringing or calling pedestrians who do not notice your presence to inform them of your presence.	Signaling violation
35- Riding a bicycle in the blind spot of cars (so that the driver does not notice you).	Traffic Violations
36- Grabbing the back of the car while riding a bicycle to move forward.	Stunts/Distractions
37- Riding a bicycle without hands or with one hand.	Stunts/Distractions
38- Cycling at night without reflective equipment or clothing (night vision).	Traffic Violations
39- Chasing the driver, pedestrian, or cyclist who made you angry.	Traffic Violations
